# Demographic resilience of brook trout populations subjected to experimental size‐selective harvesting

**DOI:** 10.1111/eva.13478

**Published:** 2022-09-18

**Authors:** Shannon H. Clarke, Gregory R. McCracken, Shelley Humphries, Daniel E. Ruzzante, James W. A. Grant, Dylan J. Fraser

**Affiliations:** ^1^ Department of Biology Concordia University Montreal Quebec Canada; ^2^ Department of Biology Dalhousie University Halifax Nova Scotia Canada; ^3^ Lake Louise, Yoho, and Kootenay Field Unit, Parks Canada Radium Hot Springs British Columbia Canada

**Keywords:** effective population size, fisheries management, genetic compensation, population genetics

## Abstract

Sustainable management of exploited populations benefits from integrating demographic and genetic considerations into assessments, as both play a role in determining harvest yields and population persistence. This is especially important in populations subject to size‐selective harvest, because size selective harvesting has the potential to result in significant demographic, life‐history, and genetic changes. We investigated harvest‐induced changes in the effective number of breeders (${\hat{N}}_{b}$) for introduced brook trout populations (*Salvelinus fontinalis*) in alpine lakes from western Canada. Three populations were subject to 3 years of size‐selective harvesting, while three control populations experienced no harvest. The ${\hat{N}}_{c}$ decreased consistently across all harvested populations (on average 60.8%) but fluctuated in control populations. There were no consistent changes in ${\hat{N}}_{b}$ between control or harvest populations, but one harvest population experienced a decrease in ${\hat{N}}_{b}$ of 63.2%. The ${\hat{N}}_{b}$/${\hat{N}}_{c}$ ratio increased consistently across harvest lakes; however we found no evidence of genetic compensation (where variance in reproductive success decreases at lower abundance) based on changes in family evenness ($\hat{\mathrm{FE}}$) and the number of full‐sibling families (${\hat{N}}_{\mathrm{fam}}$). We found no relationship between $\hat{\mathrm{FE}}$ and ${\hat{N}}_{c}$ or between ${\hat{N}}_{\mathrm{fam}}$/${\hat{N}}_{c}$ and $\hat{\mathrm{FE}}$. We posit that change in ${\hat{N}}_{b}$ was buffered by constraints on breeding habitat prior to harvest, such that the same number of breeding sites were occupied before and after harvest. These results suggest that effective size in harvested populations may be resilient to considerable changes in N_c_ in the short‐term, but it is still important to monitor exploited populations to assess the risk of inbreeding and ensure their long‐term survival.

## 1 INTRODUCTION

An ongoing concern in fisheries and wildlife management is ensuring the sustainability of both commercial and recreational harvest. Selective pressures introduced through harvest can drive evolutionary and ecological changes, and potentially reduce harvest yields and wild population persistence (Fraser, 2013; Kuparinen & Festa‐Bianchet, 2017). One of the most common harvest strategies in fisheries is size‐selective harvest, where the largest individuals are targeted, generating selection on body size (Law, 2000; Stokes & Law, 2000). Size‐selective harvest often results in considerable life history changes, including earlier maturation, reduction in body size, and reduced fecundity (Fenberg & Roy, 2008; Kuparinen & Merilä, 2007). Demographic processes also play a role in determining the effects of size‐selective harvesting on exploited populations; processes such as density‐dependence, competitive release, or Allee effects may influence how populations respond to or recover from harvest (Gobin et al., 2016; Kuparinen et al., 2014; Zipkin et al., 2008). Recent studies have also highlighted the importance of tracking genetic change in exploited populations in order to predict long‐term viability and ensure sustainable management (Allendorf et al., 2008; Ovenden et al., 2015). Therefore, monitoring both genetic and demographic processes can play an important role in managing populations exploited by size‐selective harvesting.

One important genetic parameter for evaluating threats to wild populations is the effective population size (*N*_*e*_); it integrates both life history and genetic changes and can track inbreeding and genetic drift in populations (Waples, 2002). Populations with smaller *N*_*e*_ are at higher risk of losing genetic variation through drift than larger populations. In managed populations, *N*_*e*_ is often considered alongside the demographic population size, or census size (*N*_*c*_), in order to combine both demographic changes and genetic processes into a single metric. The ratio of *N*_*e*_ */N*_*c*_ in an idealized population should be ~1, because all individuals should have equal chance at contributing to successive generations; however, in natural populations, ${\hat{N}}_{e}$ (with the hat referring to an estimate, rather than the parameter *N*_*e*_) is often much lower than ${\hat{N}}_{c}$ (Palstra & Fraser, 2012; Palstra & Ruzzante, 2008; Ruzzante et al., 2016). The factors that drive ${\hat{N}}_{e}$ below ${\hat{N}}_{c}$ include unequal sex ratios, variance in reproductive success, and fluctuations in *N*_*c*_ (Franklin, 1980); all of which may occur in exploited populations. Estimating *N*_*e*_ in wild populations, however, can be difficult due to the need for demographic data from a species' entire lifespan, or the need for multiple temporal genetic samples (Waples et al., 2014). Instead, many studies use the effective number of breeders (*N*_*b*_), which is a measure of effective size for a single reproductive season, making it a much more accessible parameter than *N*_*e*_ (Ferchaud et al., 2016). *N*_*b*_ is closely linked with *N*_*e*_ through life‐history traits (Waples et al., 2013), and is also often compared alongside *N*_*c*_ to evaluate both demographic and genetic changes in managed populations (Palstra & Fraser, 2012).

The ratio between ${\hat{N}}_{b}$ and ${\hat{N}}_{c}$ may differ between populations of the same species due to differences in population size, environmental variation, or different life history characteristics. Larger populations generally have a higher ${\hat{N}}_{b}$ than smaller populations (Yates et al., 2017); however, density‐dependent effects on reproduction often lead to very large populations having a smaller ${\hat{N}}_{b}$/${\hat{N}}_{c}$ ratio than small populations (Bernos & Fraser, 2016; Ferchaud et al., 2016). In addition, environmental variation may influence ${\hat{N}}_{b}$/${\hat{N}}_{c}$ in species with specific breeding or habitat requirements (Shrimpton & Heath, 2003; Whiteley et al., 2015). Finally, life history characteristics such as body size, age at maturity, and mating systems can also impact ${\hat{N}}_{b}$/${\hat{N}}_{c}$ (Jones & Hutchings, 2001; Lee et al., 2011; Waples & Antao, 2014). Most research on these topics has been observational – not experimental – and done spatially across populations, not temporally within populations. Although the general trends found in observational studies may be representative of how demographic and genetic processes act within populations, experimental studies are still necessary to fully understand how populations may respond temporally to disturbances such as harvest.

Over the long term, intensive harvest is expected to decrease both *N*_*b*_ (by reducing the number of potential adults available to breed, e.g. Diaz et al., 2000, or by changing the variance in reproductive success) and *N*_*c*_ (by increasing mortality rates). However, ${\hat{N}}_{c}$ is likely to decrease more rapidly than ${\hat{N}}_{b}$, leading to an increase in ${\hat{N}}_{b}$/${\hat{N}}_{c}$. Many demographic factors moderate changes in *N*_*b*_ and may even lead to increases, especially in the short‐term. For example, density‐dependent effects may result in genetic compensation (i.e., where *N*_*b*_ */N*_*c*_ increases) at small population size where the variance in reproductive success decreases due to a decline in the intensity of competition for reproductive opportunities (Ardren & Kapuscinski, 2003; Bernos & Fraser, 2016). Kuparinen et al. (2016) showed that ${\hat{N}}_{c}$ decreased proportionally more than ${\hat{N}}_{e}$ during simulated harvest of cod stocks, and in some cases ${\hat{N}}_{e}$ actually increased (the impacts on *N*_*e*_ and *N*_*b*_ are expected to be similar). However, using ${\hat{N}}_{b}$/${\hat{N}}_{c}$ as an indicator for genetic compensation has some limitations, as it relates a ratio to its denominator and does not directly test for variance in reproductive success (Palstra & Ruzzante, 2008). Alternatively, family evenness ($\hat{\mathrm{FE}}$), a measure of the variance in family size, can be used as an inversely proportional measure of variance in reproductive success to test for genetic compensation in harvested populations (Whiteley et al., 2015). Size‐selectivity during harvest can also moderate changes to *N*_*b*_ through competitive release, since in some populations spawning is dominated by the largest size‐classes (Anderson et al., 2010; Blanchfield et al., 2003). Furthermore, harvest may alter *N*_*b*_ if it leads to changes in the sex ratio within a population (e.g., a skewed sex ratio can result in lower *N*_*b*_). Size‐selective harvest can alter sex ratios if the sexes differ in size, or if one sex (often male) is more vulnerable to harvest than the other (Allendorf & Hard, 2009; Kendall & Quinn, 2013). Therefore, *N*_*b*_ and *N*_*b*_ */N*_*c*_ are expected to be impacted by size‐selective harvesting through a variety of demographic and genetic processes.

Iteroparous salmonid fishes are valuable study species to observe changes in *N*_*b*_ */N*_*c*_ from size‐selective harvest. ${\hat{N}}_{b}$/${\hat{N}}_{c}$ ratios in salmonid species are well documented in the literature and can vary from <0.01 to upwards of 0.5 (Bernos et al., 2018; Ferchaud et al., 2016). Salmonids are also characterized by specific spawning habitat, and intense competition for reproductive opportunities, therefore they are very likely to be affected by density‐dependent processes (Ardren & Kapuscinski, 2003). In brook trout (*Salvelinus fontinalis*), females compete for sites of varying quality on breeding grounds (Blanchfield & Ridgway, 2005), and breeding is often skewed towards larger males (Blanchfield et al., 2003). Finally, iteroparous salmonids such as brook trout have short generation times (e.g. Gray et al., 2014; Letcher et al., 2007), which allows for shorter‐term studies on size‐selective harvesting that can span changes in ${\hat{N}}_{b}$/${\hat{N}}_{c}$ across an entire generation.

This study addresses gaps in the literature by examining how genetic (*N*_*b*_) and demographic (*N*_*c*_) variables respond to size‐selective harvest. Specifically, we asked how size‐selective harvesting influenced ${\hat{N}}_{b}$ and ${\hat{N}}_{b}$/${\hat{N}}_{c}$ in populations of brook trout in the Rocky Mountains. We predicted that ${\hat{N}}_{c}$ would decrease after successive years of intensive harvest while ${\hat{N}}_{b}$ would either be unchanged or decrease at a slower rate than ${\hat{N}}_{c}$ due to competitive release from removing the largest individuals in the population with ${\hat{N}}_{b}$/${\hat{N}}_{c}$ therefore increasing at lower population sizes (i.e., genetic compensation). The genetic compensation hypothesis further predicts that the variance in family size will decrease after harvest. We tested this hypothesis by estimating the number of offspring in each full‐sib family and estimating family evenness ($\hat{\mathrm{FE}}$). We also expected that secondary effects of harvest, such as changes to the sex ratio or reductions in body size would have an impact on ${\hat{N}}_{b}$, with decreases in ${\hat{N}}_{b}$ if the sex ratio became skewed from harvest and increases in ${\hat{N}}_{b}$ from competitive release if all larger bodied fish were removed from the lakes. Lastly, we expected that there may be population‐specific responses to harvest based on the specific demographics or life history of each population.

## 2 MATERIALS AND METHODS

### 2.1 Study system

**FIGURE 1:**
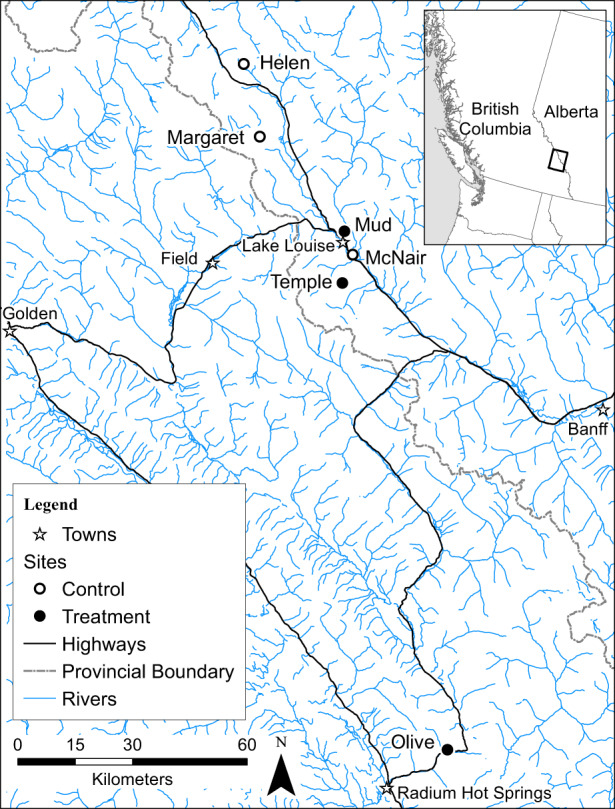
Map of six brook trout populations in alpine lakes in Banff and Kootenay National Parks in the Canadian Rocky Mountains.

The brook trout populations of interest inhabited six alpine lakes across two national parks in western Canada: Banff and Kootenay National Parks (Figure 1). These lakes were chosen due to their physical isolation, small size, fish communities dominated by brook trout, and limited inlet or outlet expanse (Table S1). Previous work in this system showed no difference in neutral genetic diversity between populations (due to their common stocking source), but showed a minor degree of adaptive genetic differentiation (Brookes et al., 2022). Although most lakes were isolated, Margaret and McNair could potentially be open to seasonal gene flow from adjacent populations through outlet waterfalls during extreme weather events (Adams et al., 2000; Thompson & Rahel, 1998). These populations spawn in the lakes, and in accessible inlets or outlets (Table S1). The populations in these lakes were assumed to be in an equilibrium state due to little or no exploitation before this project. Although Parks Canada does not record angler effort on a per‐lake basis, Helen, Mud, and Olive are accessible to the public and are known to be subject to minimal catch‐and‐release recreational fishing (Shelley Humphries, Parks Canada, personal observation). We subjected three of these lakes to size‐selective harvesting over three successive years, with an average harvest rate of 0.59 (i.e., 59% of all adult fish were harvested, beginning with the largest fish and slowly decreasing the size of harvested fish; Table S2). This harvest rate is close to those seen in some fisheries in marine and freshwater environments (Allen et al., 2008; Thomas et al., 2009; Topping & Szedlmayer, 2013). The other three lakes were left as non‐harvested controls but were monitored over the same time span.

### 2.2 Census size estimation and experimental harvest of lakes

Each field season (during the summers of 2017–2019) was subdivided into three components: marking and size estimation; stock assessment; and harvest, in that order. Firstly, the$\;{\hat{N}}_{c}$ of brook trout in each lake was obtained through mark‐recapture methods. We captured fish of all size classes using fyke nets and inserted BioMark HPT8 pre‐loaded Passive Integrated Transponder (PIT) tags into adults >80 mm (those <80 mm were categorized as juveniles), therefore our ${\hat{N}}_{c}$ estimates corresponded with the adult population (though there could have been some immature individuals included, discussed further in the caveats section). We also measured fork length (in mm) for each fish captured. On each successive visit, the number of tagged fish and untagged fish was recorded, all the PIT tag IDs of tagged fish were noted, and all new fish were also inserted with a PIT tag. We returned daily and repeated this process until the proportion of tagged fish reached ~0.25, in order to ensure an accurate population estimate. Marking took, on average, 13 days (ranging from 7 to 20), and each lake was sampled consecutively until marking was complete. All fish were handled humanely, and these methods were approved by the Concordia University Animal Research Ethics Committee, according to the Canadian Council of Animal Care guidelines (protocol number 30007841).

We used the Schnabel method to estimate ${\hat{N}}_{c}$ using multiple recapture events. This method is widely used in fisheries science and has several assumptions including: the population is closed (no emigration or immigration), the fish retain their tags PIT tag retention in salmonids is generally >95%; (Dare, 2003; Gries & Letcher, 2002; Ostrand et al., 2011), and there is an equal probability of catching all fish (Huggins & Chao, 2005). The Schnabel estimate was generated on the mark‐recapture data using the FSA package (fisheries stock assessment, version 0.8.30) in R Statistical Software (Ogle et al., 2019; R Core Team, 2021). This package generated an estimate of ${\hat{N}}_{c}$ along with upper and lower 95% confidence intervals associated with the estimate.

**FIGURE 2:**
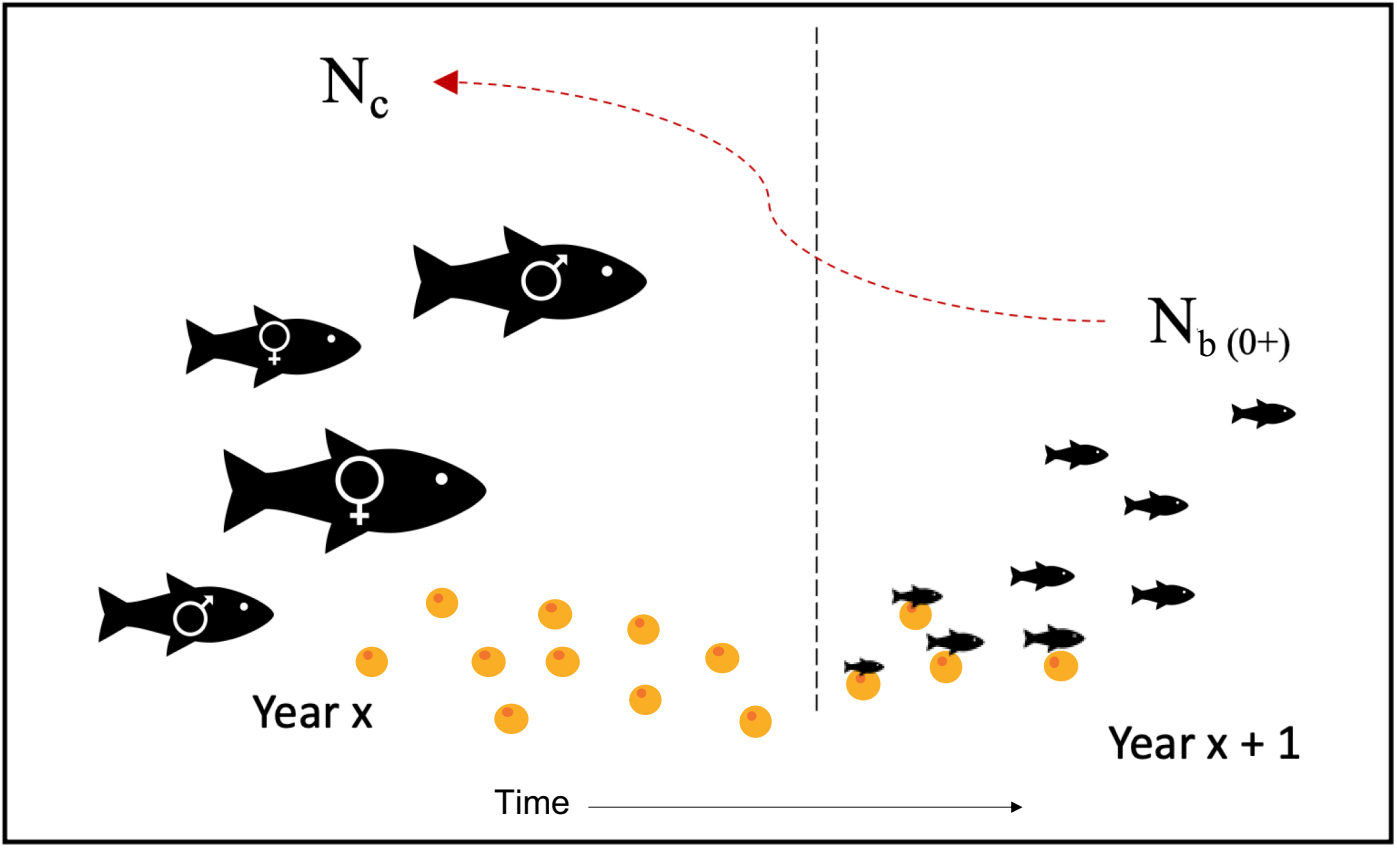
Correct linking of effective number of breeders (*N*_*b*_) to corresponding census size (*N*_*c*_) in Brook Trout, where spawning occurs in the fall and fry emerge in the following spring (with the orange dots representing the eggs). *N*_*b*_ of a 0+ (young‐of‐the‐year) cohort should be linked with the N_c_ of the adults in the previous year that produced the cohort (indicated in the figure by the dashed red arrow).

Once ${\hat{N}}_{c}$ was estimated, we conducted the stock assessment, where ~9% of the fish from both control and harvest lakes (ranging from 4% to 15%) was harvested using multi‐mesh gill nets to target all size classes. These fish were lethally sampled in order to obtain standardized size, age, and sex data, which allowed us to contrast the life histories and demographics spatially between populations and temporally within populations. This harvest rate varied due to requiring a minimum of ~30 individuals to generate demographic information on the populations, and in smaller populations (e.g., McNair or Mud) this sometimes represented a larger proportion of the population. Additionally, some days the fish were more active, and our catch was higher than expected; however, in general we tried to minimize the number of fish harvested from the control lakes to avoid inducing change in these populations. Once stock assessment was completed, we began removing individuals from the harvest lakes. The size structure within a given population was determined from length measurements from the multi‐mesh gill nets and was used to determine which mesh size of gill net was needed to target the largest individuals, while allowing the smallest ones to pass through. These gill nets were then placed in the harvest lakes and checked every 1–3 days. The gill nets were removed only when the targeted harvest rate was reached (note that the harvest rate included any individuals removed using the multi‐mesh gill nets during stock assessment). During this time the control lakes were left untouched. This harvest period occurred in late summer and early fall, before individuals were able to breed, therefore the ${\hat{N}}_{b}$ calculated in the following year should relate to only those individuals left after the harvest was completed (see Figure 2).

### 2.3 Tissue collection and microsatellite sequencing

To calculate ${\hat{N}}_{b}$ annually in each lake, young‐of‐the‐year (YOY) trout were sampled in 2017, 2019 and 2020 using a backpack electrofisher. The shoreline of each lake was electrofished, targeting fish under ~80 mm. These fish were measured (in mm), a small clip was taken from their caudal fin and placed in an Eppendorf tube with 95% ethanol, and the fish were released. We targeted a minimum of 80–100 YOY when possible. If there were insufficient numbers of YOY along the shoreline, then inlets and outlets were fished to supplement the sample size. YOY samples were taken from as many locations along the shoreline or inlet/outlets as possible, to ensure the ${\hat{N}}_{b}$ calculated from these samples was representative of the whole population (rather than a single brood). Across all lakes and years, sampling took 2.8 days and covered on average 344 m of shoreline or inlet/outlet (Table S3). Prior to sample analysis, we compared the lengths of individuals to specific length‐age curves (generated from otolith aging and sizes from individuals in these population) to ensure we only sampled YOY, and no 1+ individuals were included in the analyses. We also constructed length‐frequency histograms to differentiate YOY from 1+ individuals; these histograms were strongly bimodal, and the length cut‐offs we used varied from 50 to 75 mm (depending on the lake and timing of sampling).

When evaluating ${\hat{N}}_{b}$/${\hat{N}}_{c}$ ratios it is important that the ${\hat{N}}_{b}$ and ${\hat{N}}_{c}$ values are correctly linked – that is, that the ${\hat{N}}_{b}$ estimate from a cohort is matched with the ${\hat{N}}_{c}$ estimate from the parental generation that produced that cohort (Figure 2). The YOY samples collected in 2017 (i.e. the 0+ cohort) would therefore correspond to “pre‐harvest”, as they would have been produced by the parental generation in 2016. Because we did not have census data from 2016, the 2017 YOY samples were paired with 2017 census data, as this census data should also correspond to “pre‐harvest”. In other non‐exploited populations, Bernos and Fraser (2016) compared “incorrectly” linked ${\hat{N}}_{b}$/${\hat{N}}_{c}$ from the same year and found similar patterns as in correctly linked ratios. Because the harvest period occurred prior to spawning, when calculating the ${\hat{N}}_{b}$/${\hat{N}}_{c}$ ratio for 2019 and 2020 we accounted for any individuals removed during harvest (for treatment lakes) or during age and maturity determination (in both control and treatment lakes).

DNA was extracted from fin clips of 1248 individuals using a modified Chelex protocol (see Yue & Orban, 2005). The quality and quantity of the DNA extracts was assessed via visualization on 1% agarose gel stained with SYBR Safe (Thermo Fisher Scientific, Waltham, MA), and a Qubit fluorometer (Invitrogen). These extracts were sequenced at 33 microsatellite loci (from Ruzzante et al., 2019) using an Illumina MiSeq Sequencer along with the MEGASAT software and pipeline (Zhan et al., 2017). A single multiplex PCR consisting of all 33 loci was performed per individual using the Qiagen Multiplex PCR Kit (Qiagen Inc., Valencia, CA, USA) on Eppendorf (Hamburg, Germany) Mastercycler ep384 PCR machines. The resulting multiplex PCR products were diluted with 20 μl of purified water in preparation for indexing PCR (see Zhan et al., 2017 for details). PCR products were then pooled in equal proportion and cleaned using a 1.8:1 ratio of Sera‐Mag Speedbeads (GE Healthcare, Little Chalfront, UK) to pooled PCR library. Library quantification was completed on a Roche LC480 qPCR machine (Roche, Basel, Switzerland) using the appropriate Kapa Library Quantification Kit (*Roche, Pleasanton, California)* for use with the Illumina sequencing platform. Post sequencing, dual indexed individuals were demultiplexed automatically using the MiSeq Sequence Analysis software. This information was then input to MEGASAT (Zhan et al., 2017) which further demultiplexed individuals sequence data, based on loci, using locus specific information. Simultaneously, this software genotyped all loci, and output depth histograms for manual verification of scoring accuracy/consistency. Further details on the molecular protocol and loci can be found in Supporting Information (Table S4).

The microsatellite scoring was verified manually using the depth vs size histograms produced by MEGASAT. Microchecker (v2.2.3; Van Oosterhout et al., 2004) was used to test for potential null alleles, large allele dropout, and accidental scoring of stutter bands. Hardy–Weinberg Exact (HWE) tests for heterozygote deficiency and excess, as well as tests for linkage disequilibrium (LD) between loci were performed in GenePop (v4.7.5; Rousset, 2008). Observed and expected heterozygosity and mean number of alleles were calculated in Arlequin (v3.5.2.2; Excoffier & Lischer, 2010).

### 2.4 Estimation of effective number of breeders and family evenness

Estimates of ${\hat{N}}_{b}$ were generated using the Linkage Disequilibrium (LD) method implemented in NeEstimatorV2 (Do et al., 2014). The LD method is a single‐sample estimator first developed by Hill (1981) that uses non‐random associations of alleles at different loci (i.e., deviations from the expected genotype frequency based on random distribution) to estimate random drift and therefore *N*_*e*_ or *N*_*b*_. Here, because all individuals are from the same cohort, our estimates correspond with *N*_*b*_. The LD method implemented in NeEstimator v2 is a bias‐corrected version, that reduces downward bias when sample size is smaller than the true effective size (Waples, 2006). We used an allele frequency cutoff of 0.02, a value that balances precision and bias across sample sizes (Waples & Do, 2008), and 95% confidence intervals were generated by jackknifing across individual.

To additionally investigate the influence of harvest on family structure and genetic compensation, we used COLONY version 2.0.6.8 (Jones & Wang, 2010) to reconstruct full‐sibling families. We assumed male and female polygamy, and no sibship prior. From COLONY, we obtained estimates of the number of full sibling families (${\hat{N}}_{\mathrm{fam}}$), along with the number of individuals per family. To generate a measure of variance in family size, we estimated family evenness ($\hat{\mathrm{FE}}$) as outlined in Whiteley et al. (2013). Briefly, we used the following equation:

$$\mathrm{FE}=\frac{H^{\prime }}{H_{\mathrm{Max}}^{\prime }}$$

where $H^{\prime }=-\sum_{1}^{S}p_{i}\ln\left(p_{i}\right)$ and $H_{\mathrm{Max}}^{\prime }=\ln\left(S\right)$ (Mulder et al., 2004). *S* represents the number of families and *p*_*i*_ represents the proportion of the *i*‐th family.

### 2.5 Statistical analyses

All statistical analyses were performed in R statistical software version 4.0.5 (R Core Team, 2021). To test whether ${\hat{N}}_{b}$ and ${\hat{N}}_{c}$ were reduced through harvest, we used a two‐factor ANOVA with time (i.e. year) and treatment (harvest/control) as the predictor variables, ${\hat{N}}_{b}$ or ${\hat{N}}_{c}$ as the response variable, and lake (i.e. each population) as a random factor (random intercept). For both of these ANOVAs, if the harvest treatment had an effect on ${\hat{N}}_{b}$ or ${\hat{N}}_{c}$, we would expect to find a significant interaction between treatment and time, i.e., that over 3 years of successive harvest, the harvest lakes responded differently than the control lakes. All ANOVAs were run using the *lme* function in the *nlme* package (version 3.1; Pinheiro et al., 2017) and were tested for normality through visualization of a qqplot (using the *qqnorm* function in the *stats* package; R Core Team, 2021), as well as for homoscedasticity by plotting the normalized residuals against the fitted values. The model for ${\hat{N}}_{b}$ was log‐transformed to allow the data to approximate a normal distribution. Post‐hoc analyses were done using the *lsmeans* package (Lenth, 2018).

According to our hypothesis of genetic compensation, we expected that variance in reproductive success would decrease as ${\hat{N}}_{c}$ was reduced through harvest and as the largest and most competitive individuals were removed. Our first prediction was that ${\hat{N}}_{b}$/${\hat{N}}_{c}$ should increase in the harvest lakes, as at lower ${\hat{N}}_{c}$ there would be less competition for breeding sites, and variance in reproductive success would be lower than at higher ${\hat{N}}_{c}$. We tested for this using an ANOVA, as described above, with time, treatment, and their interaction as predictors, ${\hat{N}}_{b}$/${\hat{N}}_{c}$ as the response variable, lake as a random factor (random intercept). We log transformed ${\hat{N}}_{b}$/${\hat{N}}_{c}$ to allow the data to approximate a normal distribution and performed post‐hoc analyses using *lsmeans*.

As an additional assessment of genetic compensation, we used family evenness ($\hat{\mathrm{FE}}$), as an inversely proportional measure of variance in reproductive success in order to test two secondary predictions outlined by Whiteley et al. (2015). First, $\hat{\mathrm{FE}}$ should be negatively correlated with ${\hat{N}}_{c}$, with high evenness at low ${\hat{N}}_{c}$. Second, the ratio of the number of full‐sib families (${\hat{N}}_{\mathrm{fam}}$) to ${\hat{N}}_{c}$ should be positively correlated with $\hat{\mathrm{FE}}$, as when fewer adults produce large families, there should be greater evenness. These two predictions were tested using linear models; the first prediction was tested using a model with treatment, ${\hat{N}}_{c}$, and their interaction as predictors, $\hat{\mathrm{FE}}$ as the response, and lake as a random factor. If genetic compensation were occurring in the harvest lakes, we would expect there to be a significant interaction between ${\hat{N}}_{c}$ and treatment, with $\hat{\mathrm{FE}}$ increasing at smaller ${\hat{N}}_{c}$ in the harvest lakes. Similarly, the second prediction was tested using a model with treatment, $\hat{\mathrm{FE}}$, and their interaction as predictors, ${\hat{N}}_{\mathrm{fam}}$/${\hat{N}}_{c}$ as the response, and lake as a random factor. Based on the hypothesis of genetic compensation, we would expect to find a significant interaction between $\hat{\mathrm{FE}}$ and treatment, with ${\hat{N}}_{\mathrm{fam}}$/${\hat{N}}_{c}$ increasing at larger $\hat{\mathrm{FE}}$. These models were again run using the *lme* function and were tested for normality and homoscedasticity. Both response variables were log transformed to allow the data to approximate a normal distribution.

During size‐selective harvest, because life history traits and changes in ${\hat{N}}_{c}$ were expected to influence ${\hat{N}}_{b}$, we also tested how ${\hat{N}}_{b}$ changed with ${\hat{N}}_{c}$, sex ratio, and variance in body size using mixed models. Due to the small sample size of our dataset (*n* = 18), we could not create a full model with all of the predictor variables, so instead we created three competing models and compared their AICc to see which best explained the data. The first model included ${\hat{N}}_{c}$_,_ treatment, and their interaction as predictors; the second model included the coefficient of variation (CV) of body length (i.e. the standard deviation divided by the average), treatment, and their interaction as predictors; and the third model included the sex ratio (males/females), treatment, and their interaction as predictors. All models also included lake as a random intercept variable (random intercept models always had a lower AICc than random intercept and slope). After testing the normality of the model residuals, none of the three models conformed to the assumption of normality, therefore generalized linear mixed models were run with a gamma distribution. A gamma distribution was chosen because N_b_ values are continuous (i.e., non‐integer), and are always positive (a negative estimate indicates that there was insufficient sample size to accurately estimate *N*_*b*_). GLMMs were run using the *glmer* function in the *lme4* package (Version 1.1‐26; Bates et al., 2020), using Adaptive Gauss‐Hermite Quadrature (nAGQ = 0) in order to allow model convergence; using nAGQ = 0 does not fully account for the randomness of the random effects, but is often quite comparable with the default of nAGQ = 1, using the Laplace approximation, as it does little to lower the deviance (Bates, 2011; Stegmann et al., 2018). We also performed backward model selection, starting with the fully saturated model described above, removing variables, and comparing the AICc values between models. We chose the model with the lowest AICc, however if models were within 2 AICc of one another, we took the model with the simplest structure (i.e., the most parsimonious).

## 3 RESULTS

### 3.1 Census size estimates

**FIGURE 3:**
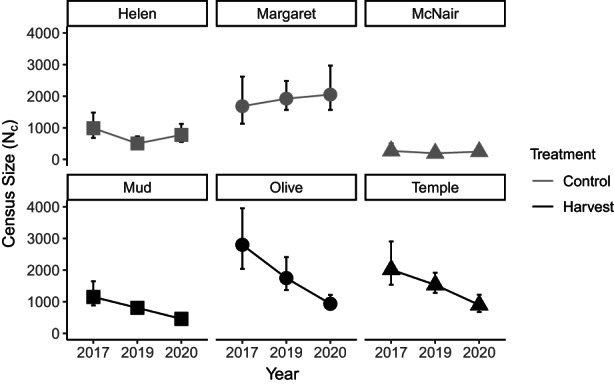
Census size (${\hat{N}}_{c}$) estimates for control and harvest brook trout populations in the Rocky Mountains from 2017 to 2019. Error bars indicate 95% confidence intervals.

Census size estimates ranged from 195 individuals (McNair Lake 2018) to 2800 individuals (Olive Lake 2017) (Table S5). All of the harvest lakes experienced a decrease in census size from 2017 to 2019 (average decline of 60.8%, ranging from 55.5% in Temple Lake to 66.6% in Olive Lake), while the control lakes fluctuated through time, or stayed relatively constant (Figure 3). The two‐way ANOVA showed that the interaction between treatment and time was significant (*p* ≤ 0.008), with the harvest lakes experiencing a significant decrease in ${\hat{N}}_{c}$ from 2017 to 2019 (*p* ≤ 0.004), and control lakes experiencing no change (*p* > 0.05).

### 3.2 Microsatellite variation

Of the 1248 individuals genotyped, 9 were removed because of missing data at 5 or more loci, and an additional 9 were removed due to evidence of sample contamination in the allele depth vs size histogram plots. One locus (ssa.27.d19) was monomorphic and was therefore excluded. Two other loci (ssa.06.8 and ssa.04.d5) were removed from subsequent analyses due to, respectively, a high percentage of ungenotyped individuals (18.8%) and a difficulty in scoring alleles at large allele sizes.

For all within‐sample microsatellite analyses, each population‐year replicate was treated as a unique “population” (i.e., Helen Lake 2017 was treated as a separate population from Helen Lake 2019 and Helen Lake 2020). Microchecker detected null alleles at locus ssa.20.d16 in a majority of the population‐year replicates (13 of 18), therefore we removed this locus from subsequent analyses. Four additional loci had null alleles detected in 2–4 of the 18 population‐year replicates, but these were spread across lakes and years, with no obvious trends. Linkage disequilibrium was detected between locus ssa‐1.7 and two other loci in 7 of the 18 population‐year replicates after Bonferroni correction, therefore locus ssa‐1.7 was removed from subsequent analyses. Only two other pairs of loci had significant linkage disequilibrium in more than one population‐year replicate, but both pairs were only significant in two of the 18 replicates, so no additional loci were removed. Only 25 out of 504 tests for heterozygote excess (<5%), and only 34 of 504 tests for heterozygote deficiency (<7%) were significant at the *p* < 0.05 level. These heterozygote excess and deficiencies were spread across all lakes and years. The final dataset therefore consisted of *n* = 1230 individuals genotyped at 28 microsatellite loci with an amplification success ≥ of 99.7%. Across all lakes and years, the average expected heterozygosity was 0.422 (0.36–0.479), average observed heterozygosity was 0.426 (0.364–0.485), and mean number of alleles per locus was 3.061 (2.364–3.429) (Table S6).

### 3.3 Effective number of breeders

**FIGURE 4:**
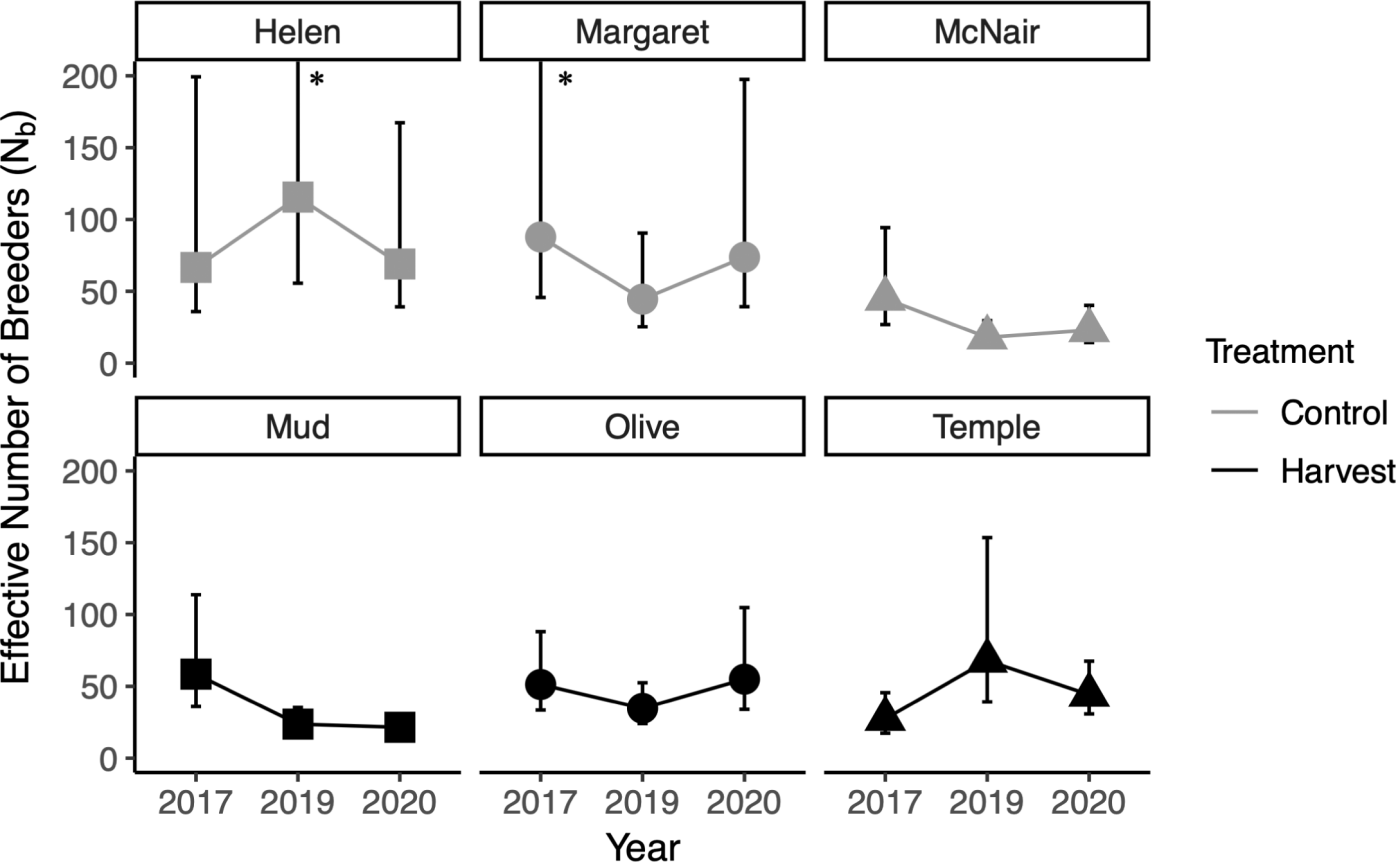
Effective number of breeders (${\hat{N}}_{b}$) change over time in control and harvest populations of brook trout in the Rocky Mountains. Error bars indicate 95% confidence intervals calculated using the jackknife method. Asterisks indicate where the error bar did not fit within the limits of the graph.

The estimates of ${\hat{N}}_{b}$ ranged from 21.5 (McNair Lake 2020) to 115.6 (Helen Lake 2019) (Table S7). All estimates had finite confidence intervals, indicating that the sample size used was sufficient in generating an accurate *N*_*b*_ estimate. The two‐way ANOVA with lake as a random effect showed no significant effect of treatment, time, or their interaction (all *p* > 0.05), indicating that ${\hat{N}}_{b}$ did not change in a predictable way in the harvest lakes compared to the control lakes. Although we did not find statistical significance in the ANOVA, we can examine the temporal pattern in ${\hat{N}}_{b}$ in each lake using the 95% CIs (Figure 4). Mud Lake was the only lake with non‐overlapping CIs through time (indicating it underwent a significant change in ${\hat{N}}_{b}$). Mud experienced a decrease in ${\hat{N}}_{b}$ from 2017 to 2019, and then ${\hat{N}}_{b}$ remained low in 2020 (no change between 2019 and 2020). All other lakes had overlapping CIs for all 3 years of sampling (Figure 4).

### 3.4 Genetic compensation

**FIGURE 5:**
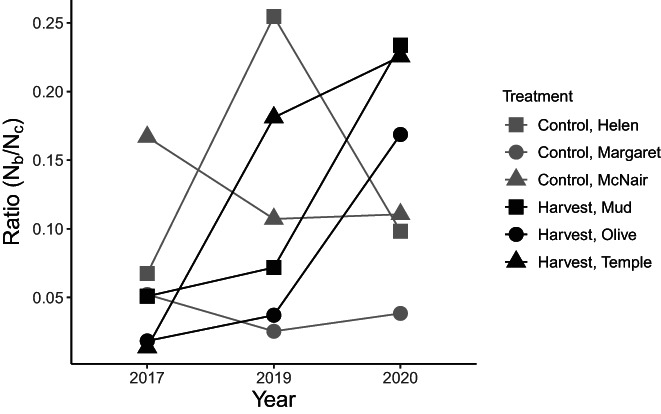
Effective to census size ratios (${\hat{N}}_{b}$/${\hat{N}}_{c}$) over time for control and harvest brook trout populations in the Rocky Mountains

Estimates of the ${\hat{N}}_{b}$/${\hat{N}}_{c}$ ratio were all well below 1, ranging from 0.014 (Temple 2017) to 0.181 (Helen 2019) (Table S8). On average, the harvest lakes experienced a 10‐fold increase in ${\hat{N}}_{b}$/${\hat{N}}_{c}$ from 2017 to 2020 (ranging from 4‐ to 16‐fold), while there was no consistent trend in the control lakes (Figure 5). The two‐way ANOVA showed a significant effect of the interaction between treatment and time (p ≤ 0.03). The standardized residuals approximated a normal distribution, although they were not heteroskedastic, indicating there are likely other predictor variables influencing ${\hat{N}}_{b}$/${\hat{N}}_{c}$ that we did not include in this model. The post‐hoc analysis of the interaction term showed that ${\hat{N}}_{b}$/${\hat{N}}_{c}$ in harvest lakes increased between 2017 and 2020 (*p* ≤ 0.017), but there was no change in the control lakes (*p* > 0.05). Therefore, consistent with our first prediction of genetic compensation, we found increased ${\hat{N}}_{b}$/${\hat{N}}_{c}$ through time in the harvest lakes only.

**FIGURE 6:**
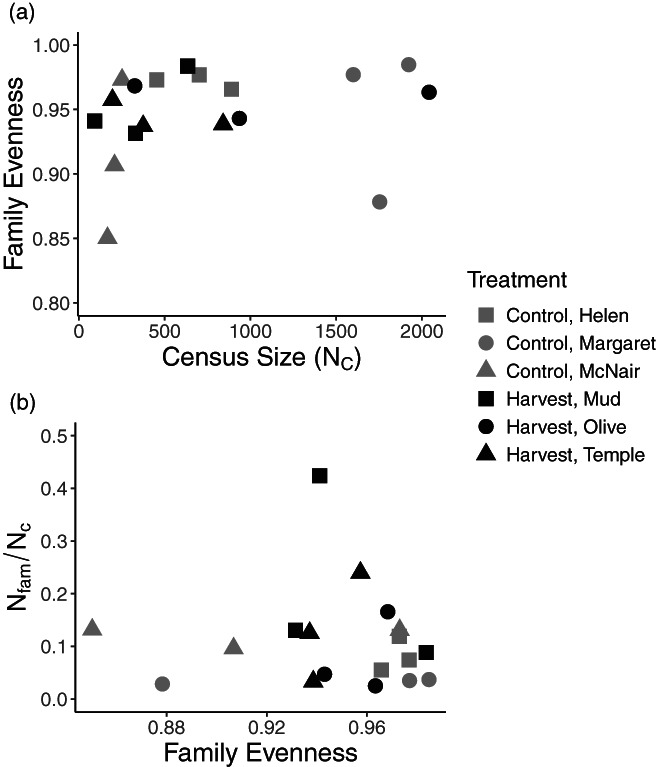
Tests for genetic compensation based on decreased variance in reproductive success between individuals. (a) The relationship between family evenness ($\hat{\mathrm{FE}}$) and ${\hat{N}}_{c}$, and (b) the relationship between the ratio of the number of full‐sib families to census size (${\hat{N}}_{\mathrm{fam}}$/${\hat{N}}_{c}$) and $\hat{\mathrm{FE}}$.

However, we did not find any evidence for genetic compensation based on the predictions for $\hat{\mathrm{FE}}$ to increase with declining ${\hat{N}}_{c}$, or ${\hat{N}}_{\mathrm{fam}}$/${\hat{N}}_{c}$ to be positively correlated with $\hat{\mathrm{FE}}$. Values of $\hat{\mathrm{FE}}$ ranged from 0.85 (McNair 2019) to 0.98 (Helen 2020, Margaret 2017 and 2020, and Mud 2017; Table S9). Values of ${\hat{N}}_{\mathrm{fam}}$/${\hat{N}}_{c}$ ranged from 0.02 (Olive 2017) to 0.42 (Mud 2020; Table S10). There were no significant terms in either of the models used to test these two predictions; there was no correlation between $\hat{\mathrm{FE}}$ and ${\hat{N}}_{c}$ (i.e. $\hat{\mathrm{FE}}$ did not increase at low ${\hat{N}}_{c}$), and no correlation between ${\hat{N}}_{\mathrm{fam}}$/${\hat{N}}_{c}$ and $\hat{\mathrm{FE}}$ (i.e. ${\hat{N}}_{\mathrm{fam}}$/${\hat{N}}_{c}$ did not increase at high $\hat{\mathrm{FE}}$) in either the control or harvest lakes (Figure 6). This indicated that the increase in ${\hat{N}}_{b}$/${\hat{N}}_{c}$ across harvest lakes cannot be attributed to genetic compensation.

### 3.5 Influence of life history on *N*_*b*_

None of the three models created had any significant terms (i.e., Treatment, N_c_, CV body length, sex ratio, and any interactions all had *p* > 0.05), and when comparing their AICc, there were no differences between the three models in their ability to predict *N*_*b*_ (the ΔAICc was <1 in all cases; Table 1). The backward model selection showed that none of the predictor variables included in the models were important, and in all cases ${\hat{N}}_{b}$ ~ 1 + (1|Lake) was always the most parsimonious model (see Table S10). Therefore, we did not find any significant influence of life history traits or *N*_*c*_ on *N*_*b*_ in these populations.

**TABLE 1:** AICc values of Generalized Linear Mixed Models comparing the influence of census size (*N*_*c*_) and life history traits on effective number of breeders (*N*_*b*_) in control and harvest alpine brook trout populations.

Model terms	Log likelihood	AICc	ΔAICc
*N*_*b*_ ~ CV body length * treatment + (1|Lake)	−79.96	179.56	0.00
*N*_*b*_ ~ sex ratio * treatment + (1|Lake)	−80.05	179.74	0.18
*N*_*b*_ ~ *N*_*c*_ * treatment + (1|Lake)	−80.41	180.46	0.90

## 4 DISCUSSION

Evaluating the impact of fisheries on both genetic and demographic variables in exploited populations is important in conservation and management, because they can be linked to one another (Bernos & Fraser, 2016; Waples et al., 2013; Yates et al., 2017). We experimentally harvested wild brook trout populations from independent lakes to assess how size‐selective harvest over three consecutive years influences *N*_*b*_ and *N*_*c*_, and whether harvest‐induced changes in demographic or life history traits resulted in a change in *N*_*b*_. Consistent with our expectations, we found that the census size rapidly decreased in all harvest lakes, and we found that ${\hat{N}}_{b}$/${\hat{N}}_{c}$ increased in the harvest lakes. However, based on secondary analyses of $\hat{\mathrm{FE}}$ and ${\hat{N}}_{\mathrm{fam}}$/${\hat{N}}_{c}$, we found no evidence that this decrease was caused by genetic compensation (i.e., we did not find that the variance in reproductive success decreased as population size decreased), therefore there were likely other density‐dependent processes influencing this relationship. Contrary to our predictions, we found no link between harvest‐induced changes in demographic and life history traits and ${\hat{N}}_{b}$. These results suggest that while demographic variables (like census size) changed rapidly after harvest, the effective size was resilient to short‐term change likely due to density‐dependent processes.

### 4.1 Demographic responses to harvest

Our data show strong support for the hypothesis that census size should decrease after 3 years of successive harvesting. It is well‐known that fishing pressure can lead to reductions in census size, as guidelines have been created for commercial fisheries associated with a maximum sustainable yield, or a maximum reduction in population size relative to their natural abundance (Froese et al., 2016). Our treatment lakes experienced a rapid decline of 60.8% over 3 years while experiencing an average annual harvest rate of ~0.59. In other natural systems, greater declines have been seen with similar or even lower harvest rates, but the change took place over a greater timespan. In the Baltic Sea, Baltic cod stocks experienced an ~80% decline over a 10‐year period (1981–1992) while the estimated mean harvest rate was 0.56 (Jonzén et al., 2001). Similarly, red grouper in the Campeche Bank declined 88% from 1986 to 2001, but fishing mortality only reached an average of 0.37 at the end of the 15‐year period (Giménez‐Hurtado et al., 2005). Therefore, while the decline in ${\hat{N}}_{c}$ in our system was markedly more rapid, it is similar to those seen in commercially harvested marine populations.

### 4.2 Harvest‐induced changes in effective size were buffered by density‐dependent processes

We found no consistent change in ${\hat{N}}_{b}$ across control or harvest lakes, and our model showed no statistical significance between control and harvest lakes. However, we did find that one harvest lake, Mud, experienced a significant decrease in ${\hat{N}}_{b}$, based on non‐overlapping CIs between 2017 and 2019. The fact that ${\hat{N}}_{b}$ did not change in at least two of the three harvest lakes agrees with our initial hypothesis, as we expected that in harvest lakes, change in ${\hat{N}}_{b}$ would be buffered by density‐dependent processes. The buffering of change in ${\hat{N}}_{b}$ is much more obvious when assessing the change in ${\hat{N}}_{b}$/${\hat{N}}_{c}$; we found that ${\hat{N}}_{b}$/${\hat{N}}_{c}$ increased in all the harvest lakes, which could indicate that genetic compensation has occurred. However, this change in ${\hat{N}}_{b}$/${\hat{N}}_{c}$ is driven by decreases in ${\hat{N}}_{c}$ (rather than increases in ${\hat{N}}_{b}$), and even if we convert our ${\hat{N}}_{b}$ estimates into generational ${\hat{N}}_{e}$, using life history trait conversions from Waples et al. (2014), there are no significant increases in effective size (Table S11 and Figure S1). Additionally, our supplementary analyses based on family evenness and family size indicate that there was no evidence for genetic compensation in either the control or harvest lakes. While our results are similar to those of Kuparinen et al. (2016) in a modelling study on Atlantic cod, where they found an increase in *N*_*e*_ */N*_*c*_ after harvest, they could not parameterize the effect, whereas here we have shown that the increase in ${\hat{N}}_{b}$/${\hat{N}}_{c}$ cannot be attributed to genetic compensation.

Genetic compensation has been described in small populations from several different species, including several salmonid populations, and is generally attributed to a lower variance in breeding success at smaller population sizes (Ardren & Kapuscinski, 2003; Beebee, 2009; Bernos & Fraser, 2016; Jehle et al., 2005; Saarinen et al., 2010; Watts et al., 2007). In salmonid populations, females often compete over high‐quality breeding sites, resulting in nest destruction or superimposition (Fleming & Reynolds, 2004). Therefore, at larger population sizes, variance in reproductive success should be larger due to competition for breeding sites (Chebanov, 1991). Although we did not find evidence of genetic compensation, i.e., variance in reproductive success did not change through harvest, the habitat constraints in these lakes may still have helped buffer change in ${\hat{N}}_{b}$. Blanchfield and Ridgeway (2005) documented a population of lake‐dwelling brook trout where breeding habitat was constrained and showed that habitat quality had a stronger influence on reproductive success than competition between individuals. If there were similar constraints on high‐quality habitat in our lakes, spawning may have been constrained both before and after harvest, and a reduction in competition resulting from the removal of individuals through harvest might not result in compensation. If spawning habitat was the limiting factor, and similar numbers of individuals were successfully spawning before and after harvest, variance in reproductive success would not be expected to change (hence the lack of change in ${\hat{N}}_{b}$). In unexploited, stream‐dwelling brook trout populations, Bernos and Fraser (2016) found a relationship between stream length, *N*_*b*_, and *N*_*c*_, with *N*_*b*_ increasing as both *N*_*c*_ and stream length increased. In a separate study, Whiteley et al. (2013) found a similar relationship with habitat patch size and *N*_*b*_ in stream‐dwelling brook trout and attributed this to the availability of spawning sites. This indicates that breeding habitat availability is likely a factor in density‐dependent processes in brook trout, especially when spawning in streams. While the populations in this study are lake‐dwelling, all of them also spawn in inlets or outlets, therefore the relationship between spawning habitat availability and *N*_*b*_ or *N*_*b*_ */N*_*c*_ is likely similar.

In addition to the competition for breeding sites, density‐dependence may also have an impact on life history traits that could result in a high *N*_*b*_ */N*_*c*_. At lower densities, individuals may have higher growth, earlier maturation, or increased fecundity (Johnston & Post, 2009; Matte et al., 2020; Rose et al., 2001), all of which could allow for the harvested populations to maintain a high *N*_*b*_ */N*_*c*_. Density‐dependent effects on *N*_*b*_ might also be population‐specific. We found differences between the three harvest lakes in their response (i.e., Mud decreased in ${\hat{N}}_{b}$ while Olive and Temple did not change), which might be attributable to different density‐dependent responses. Matte et al. (2020) showed that in brook trout populations, density‐dependent responses in growth and mortality varied, even after accounting for environmental differences. Although we could not determine the underlying mechanisms, density‐dependent processes likely played a role in buffering change in *N*_*b*_ in the harvest lakes and could allow for these populations to better adapt to future changes and increase their long‐term viability.

### 4.3 Life history traits and other influences on *N*_*b*_

Contrary to our prediction, we did not find any evidence that the sex ratio or CV of body size had an influence on ${\hat{N}}_{b}$ in these populations. In another salmonid species, the brown trout, Serbezov et al. (2012) found that unequal sex ratios did not have a large impact on *N*_*b*_, similar to what we found here. Our predictions were based on a priori knowledge that an uneven sex ratio usually leads to a reduction in effective size (Franklin, 1980), and that populations with a wider range in body size may benefit from competitive release if the largest individuals are removed. However, we did not consider whether other life‐history traits might be more important in predicting *N*_*e*_, especially in the context of exploited populations. Waples et al. (2013) showed that age at maturity and adult lifespan explain up to half of the variance in *N*_*e*_ */N*_*c*_ across 63 iteroparous species (including two salmonid species). In the context of size‐selective harvest, age at maturity and adult lifespan would also make sense as potential drivers of change in *N*_*b*_, as these two life‐history traits will likely be impacted by harvest (Enberg et al., 2012; Law, 2000). Indeed, other work from our project found that as density decreased, both males and females had an earlier maturation, especially those from age 0+, 1+, and 2+ age‐classes (J. M. Matte, unpublished data). Therefore, age‐at‐maturity may have helped to predict changes in effective size, however it may not have resulted in significant results, simply due to the lack of change in ${\hat{N}}_{b}$ across control and harvest lakes in our study. Matte et al. also looked at changes in size‐frequency distribution of body length (a visual to the CV of body length, but with more nuance). They found that two of the harvest lakes in this study, Temple and Olive, were stunted populations with low mean and variance in body size and did not experience much change in body size variance, whereas Mud decreased in variance and body sizes became smaller. While we did not find a statistical influence of variance in body size (perhaps due to sample size limitations), the results from Matte et al. may help explain why Mud was the only harvest lake in this study to experience a decrease in ${\hat{N}}_{b}$.

Two final factors that may have influenced the trends seen in ${\hat{N}}_{b}$ across our study lakes were habitat characteristics and environmental variation. Habitat characteristics such as lake size or inflow/outflow rates may have had an impact on *N*_*b*_ due to the aforementioned relationship between spawning habitat availability and *N*_*b*_. Indeed, Whiteley et al. (2015) and Bernos and Fraser (2016) showed how stream flow influenced ${\hat{N}}_{b}$, likely due to increasing habitat availability. Other environmental variables such as air temperature and precipitation may have also influenced *N*_*b*_ through secondary effects on spawning and juvenile survival (Kanno et al., 2016). While our three harvest lakes were all located within 100 km of one another, they differed in their elevation, depth, and surface area (Table S3), all of which may have influenced the available spawning habitat and environmental variables these lakes experienced. Therefore, although we did not find an influence of sex ratio or change in body size on N_b_, there were likely other factors such as habitat characteristics or environmental variation influencing the effective size.

### 4.4 Caveats and future research directions

Studies like ours that experimentally harvest wild populations with proper controls are exceedingly rare and sorely needed to inform fisheries management (Audzijonyte et al., 2013; Heino et al., 2015; Kuparinen & Merilä, 2007). Nevertheless, our work has a few caveats that are important to take into account when interpreting the results. Firstly, despite 3‐ or 4‐month long field seasons with 4+ crew members, the replication in this study was relatively low. Three years of data from six lakes only yielded 18 data points for ${\hat{N}}_{b}$ and ${\hat{N}}_{c}$, therefore we had low power to test for significance in our models. This lack of power could explain why we did not detectany statistical effect of harvest on ${\hat{N}}_{b}$ or find a link between life history traits and ${\hat{N}}_{b}$, however in this discussion we are careful to limit our conclusions to only what the data show us. Related to this replication issue is that our work was conducted over a relatively short temporal scale. There could be a time‐lag in detecting changes in *N*_*b*_, especially if density‐dependent processes only buffer change in the short‐term. If *N*_*c*_ continued to decrease in these populations, it is possible they could experience inbreeding, as the likelihood of mating with related individuals would be higher at small population sizes, and as inbreeding increased, they would subsequently experience a decrease in effective size (Wang et al., 2002). Furthermore, our results are likely from a more general consequence of increased mortality, rather than from size‐selectivity of fishing (Garcia et al., 2012). Future work would benefit from comparing balanced harvest (i.e., harvesting across all size classes) to size‐selective harvest to compare impacts on effective size, and ideally increase replication over both time and space to increase statistical power. Finally, our ${\hat{N}}_{c}$ estimates may represent an intermediate between the adult census size and the total census size for these populations. We chose a cut‐off of 80 mm for our mark‐recapture methods in order to reduce the likelihood of tag loss or death, and because we did not have prior information about size‐at‐maturity in these lakes. It is possible that some individuals of 80 mm in length were not mature. However, we suspect our methods included at most few immature individuals in calculations of ${\hat{N}}_{c}$, because tagging of fish occurred in June and early July, leaving substantial time for further growth before spawning in late September or October. The effect of having a few immature individuals included in the sampling would result in a small to modest overinflation of *N*_*c*_ estimates; however, this would also likely have little impact on our overall result of higher *N*_*b*_/*N*_*c*_ ratios in harvest lakes, for such an overinflation in N_c_ would imply that true *N*_*b*_/*N*_*c*_ ratios might actually be even higher. Therefore, we do not expect having an 80‐mm size cutoff had a significant impact on our results.

## 5 CONCLUSIONS

Assessing the impact of fisheries on the effective size of exploited populations can reveal important impacts on both demographic and genetic processes. Here, we show how in populations of size‐selectively harvested brook trout, that demographic parameters changed rapidly, while the effective size experienced little or no change. Over the short‐term, change in effective size is likely buffered by density‐dependent processes, but this does not mean that the populations are immune to decreases in *N*_*b*_ (as seen in Mud Lake). Even though *N*_*b*_ */N*_*c*_ may increase in a harvested population, it could still experience a decrease in effective size, increasing the risk of inbreeding, and reducing the fitness of the population (Charlesworth & Willis, 2009). To our knowledge, this study is also the first to show the effects of harvest in natural populations with relatively small effective and census sizes. We showed similar results to the modelling study by Kuparinen et al. (2016), however our effective sizes were much lower (ranging from ${\hat{N}}_{b}$ of ~20 to 65 in harvest lakes which would translate into *N*_*e*_ of <100 to a few hundred, compared to *N*_*e*_ of ~500 to 2000 in the modelling study), and our populations were in a lacustrine rather than a marine environment. In the context of conservation and management, showing these relationships at small population sizes is especially important, as small populations are often highly managed, and may be at a high risk of extirpation (Lande, 1993; Meuwissen, 2009). In sum, the effective size of harvested populations can be resilient to change in the short‐term due to density‐dependent processes, however proper monitoring and management of fisheries is still important to ensure the sustainability of fisheries, especially in the long‐term.

## CONFLICT OF INTEREST

The authors declare no conflict of interest.

## Supporting information


Supporting information S1

## Data Availability

The data that support the findings of this study are available from the corresponding author upon reasonable request.
