# Supplementary material for: Demographic resilience of brook trout populations subjected to experimental size‐selective harvesting
Source: Evol Appl. 2022 Sep 18;15(11):1792–805. doi: 10.1111/eva.13478 (PMC9679253; doi:10.1111/eva.13478)
Supplement: Supplementary file 1 — Supporting information S1 [file EVA-15-1792-s001.docx]

**Supporting Information**

**Table S1:** Environmental characteristics of six alpine lakes inhabited by brook trout populations in the Rocky Mountains

| Population | Treatment | Species* | Surface area (ha.) | Elevation (m) | Maximum Depth (m) | L50  (mm) | Inlet or Outlet Spawning? |
| --- | --- | --- | --- | --- | --- | --- | --- |
| Helen | Control | 1 | 2.5 | 2400 | 15.0 | 138 | Outlet |
| Margaret | Control | 1,3 | 18.0 | 1808 | 28.2 | 184 | Outlet |
| McNair | Control | 1 | 1.7 | 1532 | 4.0 | 250 | Inlet |
| Mud | Harvest | 1,2 | 7.2 | 1600 | 7.2 | 172 | Inlet |
| Olive | Harvest | 1 | 1.7 | 1470 | 3.6 | 163 | Outlet |
| Temple | Harvest | 1 | 3.3 | 2207 | 14.7 | 168 | Outlet |

*Species – 1: Brook Trout, 2: Longnose Dace, 3: Westslope Cutthroat Trout
L50: length-at-50-maturity (mm) in 2017

**Table S2**: Number of fish harvested, harvest rates (in brackets), and size range of harvested fish across experimentally harvested populations of brook trout in the Rocky Mountains

| Lake | | 2017 | | 2018 | | 2019 | |
| --- | --- | --- | --- | --- | --- | --- | --- |
|  |  | # of fish (harvest rate) | Size range (mm) | # of fish (harvest rate) | Size range (mm) | # of fish (harvest rate) | Size range (mm) |
| Harvest | Mud | 512 (0.45) | 425 - 129 | 487 (0.61) | 399 - 117 | 382 (0.84) | 350 - 102 |
|  | Olive | 788 (0.28) | 225 - 104 | 818 (0.47) | 249 - 100 | 610 (0.65) | 228 - 94 |
|  | Temple | 1159 (0.58) | 299 - 120 | 1159 (0.76) | 233 - 104 | 700 (0.78) | 233 - 102 |

**Table S3**: Sampling information for fyke netting and electrofishing. Number of sampling days for fyke netting, distance along shoreline and in inlets or outlets that were elecrofished for young-of-the-year, along with the number of sampling days for electrofishing.

| Population - Year | Number of fyke sampling days | Distance electrofished (m) | Number of electrofishing sampling days |
| --- | --- | --- | --- |
| Helen 2017 | 12 | 230 | 3 |
| Helen 2018 | 12 | 320 | 2 |
| Helen 2019 | 8 | 320 | 2 |
| Marg 2017 | 14 | 345 | 3 |
| Marg 2018 | 19 | 290 | 4 |
| Marg 2019 | 12 | 230 | 1 |
| McNair 2017 | 10 | 185 | 1 |
| McNair 2018 | 12 | 295 | 5 |
| McNair 2019 | 18 | 220 | 3 |
| Mud 2017 | 12 | 300 | 4 |
| Mud 2018 | 18 | 240 | 3 |
| Mud 2019 | 8 | 315 | 1 |
| Olive 2017 | 15 | 550 | 3 |
| Olive 2018 | 21 | 350 | 4 |
| Olive 2019 | 13 | 300 | 4 |
| Temple 2017 | 10 | 750 | 5 |
| Temple 2018 | 18 | 370 | 1 |
| Temple 2019 | 17 | 580 | 2 |

**Molecular Protocol**

Forward and reverse primers for each one of the 33 loci were purchased from Integrated DNA technologies (IDT, Coralville, IA, USA) tailed with Illumina (San Diego CA, USA) Read1__(CCCTACACGACGCTCTTCCGATCT)_ and Read2__(GTTCAGACGTGTGCTCTTCCGATCT)_ sequencing primers, respectively. A single multiplex PCR consisting of all 33 loci was performed per individual using the Qiagen Multiplex PCR Kit (Qiagen Inc., Valencia, CA, USA) following the manufacturers recommendations, scaled back to 5µl total reaction volume. The multiplex PCRs were carried out on Eppendorf (Hamburg, Germany) Mastercycler ep384 PCR machines with the following parameters: 94°C for 15m, 20 cycles of 94°C for 30s, 57°C for 180s, 72°C for 60s, and a final extension at 68°C for 30 minutes.

The resulting multiplex PCR products were diluted with 20µl of purified water in preparation for indexing PCR (see Zhan et al*.,* 2017 for details). Indexing PCRs were carried in 5µl reaction volumes consisting of 1.95µl of purified water, 0.5µl of 10x buffer, 0.2 mM of each dNTP, 0.2µM of each index oligo, 0.5µl of diluted multiplex PCR product, and 0.25U of TSG (Bio Basic, Markham, ON, Canada). The following thermocycler parameters were used: 95°C for 120s, 20 cycles of 95°C for 20s, 60°C for 60s, 72°C for 60s, and a 72°C final extension for 10 minutes.

PCR products were then pooled in equal proportion and cleaned using a 1.8:1 ratio of Sera-Mag Speedbeads (GE Healthcare, Little Chalfront, UK) to pooled PCR library. Library quantification was completed on a Roche LC480 qPCR machine (Roche, Basel, Switzerland) using the appropriate Kapa Library Quantification Kit (Roche, Pleasanton, California) for use with the Illumina sequencing platform. We followed the manufacturer’s guidelines for this quantification. Libraries were subsequently diluted to 15pM and sequenced in a single direction using an Illumina MiSeq Benchtop Sequencer (Illumina, San Diego, CA, USA). Due to expected fragment size, we utilized MiSeq 150 cycle V3 chemistry kits with dual indexing, and sequenced 150bp in one direction, rather than paired end sequencing.

Post sequencing, dual indexed individuals were demultiplexed automatically using the MiSeq Sequence Analysis software. This resulted in the output of a single FASTQ file per individual, containing sequence data for all microsatellite loci related to that individual. This information was then input to MEGASAT (Zhan et al., 2017) which further demultiplexed individuals sequence data, based on loci, using locus specific information. Simultaneously, this software genotyped all loci, and output depth histograms for manual verification of scoring accuracy/consistency.

**Table S4**: Reference information on 33 loci used in microsatellite analyses.

| Locus name | Reference | No tail left | Rev. comp of no tail right | Five Flank | Three Flank | Repeat |
| --- | --- | --- | --- | --- | --- | --- |
| SFOC88 | King et al. (2012)^1^ | GGGAGAACCCAGTGTTTCTTT | CGTTCACAATCAGGGTTCAG | ATTTGACCTAAGTGAACTGTGTTATAAG | GA | GAT |
| SFOC24 | King et al. (2012)^1^ | AACACTGGAGCCGTTGAAGT | TTAGGCATCACCCCATCTCT | TATGGGT | G | GAT |
| SFOD129 | King et al. (2012)^1^ | GTGCAGGCACTAACTGGACA | TGAAGATGAGGATTCCCTGG | GAGCCTGAGGACCCTGAGGATGAAGATGACGTTCCT | GAAGAGGACCCTCTTATATT | GAT |
| SFOC28 | King et al. (2012)^1^ | CAGTTGAAGTGATTGGGTTAGC | GCTTTTGTGTGTGTGGTGTG | TACTTCTGTTTCTTTCTCTGTGTGTGTGT | GC | GCGT |
| SFOC113 | King et al. (2012)^1^ | GGGGAGCCCAGACTATATTGA | TACCCTGATGGCAATGATGA | CGATGACAACTACATAGACAGT | GAA | GAT |
| Ssa-1.14 | Bradbury et al. (2018)^2^ | TCGTATTTGTCAAGGATGTGCC | GGGCAATACAATGGGCATCT | TTG | TTTATT | AGT |
| Ssa-1.7 | Bradbury et al. (2018)^2^ | AGAACACAACAGAACCAGGTAC | GGGTTGGAAGTGTGTTCGAG | TACTGACCATGGTCCT | GGTCAATAATCCCTCACACTG | GAT |
| Ssa-10.2 | Bradbury et al. (2018)^2^ | TGATCCTCTTCACCACCCTG | GGTGAGGGAGGAGTCTTCAG | TGTTCTCCACAT | ATCAT | AAT |
| Ssa-15.1 | Bradbury et al. (2018)^2^ | TTTCTTTGTGTGTTGTGCCC | CCCAGAGGAACCACAGCTG | TTTAATCATC | CAGTTGTCCCTGGC | CCT |
| Ssa-21.5 | Bradbury et al. (2018)^2^ | CACTCCCTAACTCCATGGTC | CACAGTGACGACATCCATGA | ATCACATGGTCCTAGTACTGAACGGGAAAG | AAAGGACAAACGCAAATAGAGGACGT | GAT |
| Ssa-26.d06 | Bradbury et al. (2018)^2^ | CATAATCACCTTGCATGACACC | GTATTTAGCGGTGCAGCAGG | TTTAT | CT | AC |
| Ssa-01.12 | Ian Paterson, unpublished | ACAATGTCGCT | GTAATCGGCGGCTACAGATG | GCTGTACT | GGATCTCCATGTGGTC | GCT |
| Ssa-05.10 | Ian Paterson, unpublished | GCTTCCACGCCCATAACAAC | GAGAGCCGAGGAGTTGGAG | AACATGGC | GGTGGAGAATAACAAC | AGG |
| Ssa-03.7 | Lien et al. (2016)^3^ | GCACATTGAAGTTGGTTGCC | GACGAGCAGCTTCTGGTTAA | AACCACCATATAAAAACCA | X | AAG |
| Ssa-04.d56 | Lien et al. (2016)^3^ | CTGCTGGTAAATGGGCGTTG | GCCTTTGTTAACCAGGACAGG | GTGCA | TTTGAA | GT |
| Ssa-06.8 | Lien et al. (2016)^3^ | TGAGGCCGATGTCACCTG | GCCGTCGAGTTCAGGTACT | GATGTGACCTGTCACCGTCATC | CCCTGTTCGGAAT | CCT |
| Ssa-09.12 | Lien et al. (2016)^3^ | CTGACAGGTGGAGTGGGAC | CTGCAGGTACATGCGGGA | CCCTCTCCATC | GTTCGTCTGG | TCC |
| Ssa-10.3 | Lien et al. (2016)^3^ | TGATGGGTCTTGGTGTAGGG | CCTGGGCTTCACCGTTGA | GTGATAGA | GGTGGTCTCTCCCTGGCTCTGGC | AGT |
| Ssa-11.1 | Lien et al. (2016)^3^ | AGAGCTCCGACACACATTCG | GCACCGGCCTAGCTCTATG | GCCG | CAGGCAGCACTGCATTTTTGA | CCT |
| Ssa-12.2 | Lien et al. (2016)^3^ | ACTGGTAGGTCATTGTTCTGTG | GCACCGAGAACACACATAAGG | TTTTGATCAGCACACATTATTTTTTATTTTATTACTATT | CT | ACT |
| Ssa-13.6 | Lien et al. (2016)^3^ | GCTGTTCCTCTGGCCTCAC | GATAGTACAGTGTTGAGGTGCT | CTCCTTGT | TAGTCTGGTCA | TCC |
| Ssa-14.10 | Lien et al. (2016)^3^ | GGGAACGTGTGGAAGATTCAC | GGCATCACCCTCCATACCTT | TCACTAAGGTCCTC | AGC | ATC |
| Ssa-15.9 | Lien et al. (2016)^3^ | ATACTACCTGTTCAGGCGGC | GGGAGGAGAGTCATCACAGG | CACCGG | A | AGG |
| Ssa-16.2 | Lien et al. (2016)^3^ | GTTTACGTCACCTGCAGCTG | CGAGGGCTTAACATCTACTGC | AGCCCCCTCCACTCT | ACCACCACTA | ACT |
| Ssa-20.3 | Lien et al. (2016)^3^ | GGAGGGAGTGTAGAGGCTTTC | GTAGCAGAGATGGGTGTGTG | AGTTGAATTGTTTACCACG | T | AGG |
| Ssa-20.d16 | Lien et al. (2016)^3^ | GGCAACGAGGTGAGAATGC | GGCAACTAGGTAAGACGCAC | GC | GGTCA | AC |
| Ssa-23.9 | Lien et al. (2016)^3^ | ACGGATACAGAGAGACGCAC | GACTTTGTCCTCCTCGCTGT | GCGGGATATACTAGTGTTATCAGTATTTTCTTT | AAGGTGGTGGTATCACCTTTAG | ATC |
| Ssa-27.1 | Lien et al. (2016)^3^ | TCCATGAGTACACGCCACTG | CCAGGGTAGAGTAGTGGAGAAC | ACT | X | CT |
| Ssa-27.d07 | Lien et al. (2016)^3^ | GATTTCACAAAGCAGCGCG | CTCGCGACAGAACATGCTG | CGCCAGTGTACC | GCGGGTC | AC |
| Ssa-27.d19 | Lien et al. (2016)^3^ | GGAATACTGTCTCATTGCGCC | CACCTACAACCTTTGATTGCCT | TCAAGTGTGACT | CGAGAGATTA | GT |
| Ssa-28.d08 | Lien et al. (2016)^3^ | TCTGACCTACACACAACAATGG | CATTCTGAGCGAGCACACAC | AAGAGCTA | AAACA | AC |
| Ssa-29.2 | Lien et al. (2016)^3^ | GGCACAGCACACCAGTTG | GGAACATCTTGGAACGCTGT | TCTCTCCTTCTCTTCT | CACTCCTCTCATCTGGTCTTCTTTA | TCC |
| Ssa-4.9 | Lien et al. (2016)^3^ | AGAATCTCTAGCCCACACAAC | GCTCACATCTCAACCCTGC | X | TA | AAC |

^1^King, T. L., Lubinski, B. A., Burnham-Curtis, M. K., Stott, W., Morgan, R. P. (2012). Tools for the management and conservation of genetic diversity in brook trout (Salvelinus fontinalis): tri-and tetranucleotide microsatellite markers for the assessment of genetic diversity, phylogeography, and historical demographics. *Conservation Genetics Resources*, 4(3), 539-543.

^2^Bradbury, I. R., Wringe, B. F., Watson, B., Paterson, I., Horne, J., Beiko, R., Lehnert, S. J., Clement, M., Anderson, E. C., Jeffrey, N. W., Duffy, S., Sylvester, E., Robertson, M., Bentzen, P. (2018). Genotyping‐by‐sequencing of genome‐wide microsatellite loci reveals fine‐scale harvest composition in a coastal Atlantic salmon fishery. *Evolutionary Applications*, 11(6), 918-930.

^3^Lien, S., Koop, B. F., Sandve, S. R., Miller, J. R., Kent, M. P., Nome, T., … Davidson, W. S. (2016). The Atlantic salmon genome provides insights into rediploidization. *Nature*, 533(7602), 200-205.

**Table S5**: Census size (*N_c_*) and 95% CIs over three successive years for control and harvest alpine brook trout populations

| Lake | | 2017 | | |  | 2018 | | |  | 2019 | | |
| --- | --- | --- | --- | --- | --- | --- | --- | --- | --- | --- | --- | --- |
|  |  | N_c_ | LCI | UCI |  | N_c_ | LCI | UCI |  | N_c_ | LCI | UCI |
| Control | Helen | 988 | 682 | 1484 |  | 504 | 385 | 729 |  | 777 | 553 | 1127 |
|  | Margaret | 1684 | 1129 | 2619 |  | 1923 | 1569 | 2483 |  | 2052 | 1569 | 2967 |
|  | McNair | 269 | 152 | 510 |  | 195 | 153 | 266 |  | 245 | 183 | 335 |
| Harvest | Mud | 1150 | 883 | 1648 |  | 805 | 690 | 968 |  | 456 | 387 | 557 |
|  | Olive | 2800 | 2038 | 3953 |  | 1747 | 1369 | 2413 |  | 934 | 759 | 1215 |
|  | Temple | 2012 | 1538 | 2906 |  | 1533 | 1277 | 1918 |  | 896 | 673 | 1218 |

**Table S6**: Expected and observed heterozygosity (H_e_, H_o_), and number of alleles (N_a_) for control and harvest populations of brook trout in the Rocky Mountains. N_micro_ indicates the sample size used in the microsatellite analyses

| Population - Year | N_micro_ | H_e_ | H_o_ | N_a_ |
| --- | --- | --- | --- | --- |
| Helen 2017 | 61 | 0.435 | 0.453 | 3.214 |
| Helen 2019 | 65 | 0.423 | 0.416 | 3.321 |
| Helen 2020 | 65 | 0.440 | 0.445 | 3.393 |
| Marg 2017 | 79 | 0.360 | 0.368 | 2.870 |
| Marg 2019 | 90 | 0.376 | 0.38 | 2.364 |
| Marg 2020 | 88 | 0.367 | 0.364 | 2.409 |
| McNair 2017 | 43 | 0.443 | 0.459 | 3.037 |
| McNair 2019 | 46 | 0.406 | 0.407 | 3.037 |
| McNair 2020 | 43 | 0.420 | 0.425 | 2.926 |
| Mud 2017 | 68 | 0.472 | 0.467 | 3.074 |
| Mud 2019 | 72 | 0.458 | 0.478 | 2.926 |
| Mud 2020 | 74 | 0.479 | 0.485 | 2.963 |
| Olive 2017 | 74 | 0.41 | 0.390 | 3.111 |
| Olive 2019 | 76 | 0.408 | 0.402 | 3.286 |
| Olive 2020 | 72 | 0.417 | 0.402 | 3.346 |
| Temple 2017 | 66 | 0.400 | 0.411 | 3.037 |
| Temple 2019 | 74 | 0.441 | 0.451 | 3.357 |
| Temple 2020 | 74 | 0.449 | 0.458 | 3.429 |
| Mean | 68.3 | 0.422 | 0.426 | 3.061 |

**Table S7**: Effective number of breeders (*N_b_*) and jackknife 95% CIs for control and harvest alpine brook trout populations

| Lake | | 2017 | | | 2019 | | | 2020 | | |
| --- | --- | --- | --- | --- | --- | --- | --- | --- | --- | --- |
|  |  | N_b_ | LCI | UCI | N_b_ | LCI | UCI | N_b_ | LCI | UCI |
| Control | Helen | 66.6 | 35.9 | 199.3 | 115.6 | 55.6 | 746.5 | 69.0 | 39.2 | 167.3 |
|  | Margaret | 87.8 | 45.7 | 279.8 | 44.5 | 25.3 | 90.5 | 73.8 | 39.3 | 197.5 |
|  | McNair | 44.9 | 26.9 | 94.3 | 17.9 | 11.2 | 29.6 | 23.0 | 14.3 | 40.2 |
| Harvest | Mud | 58.5 | 36.0 | 113.8 | 23.7 | 16.2 | 35.3 | 21.5 | 16.0 | 29.0 |
|  | Olive | 51.3 | 33.5 | 88.1 | 34.7 | 24.1 | 52.5 | 55.0 | 34.0 | 104.8 |
|  | Temple | 27.4 | 17.4 | 45.6 | 67.9 | 39.2 | 153.6 | 44.2 | 30.8 | 67.5 |

**Table S8**: Ratio between effective and census size estimates (*N_b_/N_c_*) in control and harvest alpine brook trout populations

| Lake | | 2017 | 2019 | 2020 |
| --- | --- | --- | --- | --- |
| Control | Helen | 0.067 | 0.255 | 0.098 |
|  | Margaret | 0.052 | 0.025 | 0.038 |
|  | McNair | 0.167 | 0.107 | 0.111 |
| Harvest | Mud | 0.051 | 0.072 | 0.234 |
|  | Olive | 0.018 | 0.037 | 0.169 |
|  | Temple | 0.014 | 0.181 | 0.226 |

**Table S9**: Estimates of the number of families (*N_fam_*), family evenness (FE), and ratio of *N_f_* to *N_c_* for control and harvest alpine brook trout populations.

| Population - Year | N_fam_ | FE | N_f_/N_c_ |
| --- | --- | --- | --- |
| Helen 2017 | 49 | 0.97 | 0.06 |
| Helen 2019 | 54 | 0.97 | 0.12 |
| Helen 2020 | 52 | 0.98 | 0.07 |
| Marg 2017 | 56 | 0.98 | 0.04 |
| Marg 2019 | 50 | 0.88 | 0.03 |
| Marg 2020 | 71 | 0.98 | 0.04 |
| McNair 2017 | 33 | 0.97 | 0.13 |
| McNair 2019 | 22 | 0.85 | 0.13 |
| McNair 2020 | 20 | 0.91 | 0.10 |
| Mud 2017 | 56 | 0.98 | 0.09 |
| Mud 2019 | 43 | 0.93 | 0.13 |
| Mud 2020 | 39 | 0.94 | 0.42 |
| Olive 2017 | 51 | 0.96 | 0.02 |
| Olive 2019 | 44 | 0.94 | 0.05 |
| Olive 2020 | 54 | 0.97 | 0.17 |
| Temple 2017 | 28 | 0.94 | 0.03 |
| Temple 2019 | 47 | 0.94 | 0.13 |
| Temple 2020 | 47 | 0.96 | 0.24 |

**Table S10**: Backwards model selection for Generalized Linear Mixed Models comparing the influence of census size and life history traits on *N_b_* in control and harvest alpine brook trout populations.

| Model | Model Terms | Log Lik. | AIC | ΔAIC |
| --- | --- | --- | --- | --- |
| Census Size | N_b_ ~ 1 + (1 \| Lake) | − 80.76 | 169.23 | 0.00 |
|  | N_b_ ~ Treatment + (1 \| Lake) | − 80.57 | 172.22 | 3.00 |
|  | N_b_ ~ N_c_ + (1 \| Lake) | − 80.73 | 172.54 | 3.32 |
|  | N_b_ ~ N_c_ + Treatment + (1 \| Lake) | − 80.42 | 175.85 | 6.63 |
|  | N_b_ ~ N_c_ * Treatment + (1 \| Lake) | − 80.41 | 180.46 | 11.23 |
| CV Body Length | N_b_ ~ 1 + (1 \| Lake) | − 80.76 | 169.23 | 0.00 |
|  | N_b_ ~ CV length + (1 \| Lake) | − 80.31 | 171.70 | 2.47 |
|  | N_b_ ~ Treatment + (1 \| Lake) | − 80.57 | 172.22 | 3.00 |
|  | N_b_ ~ CV length + Treatment + (1 \| Lake) | − 79.97 | 174.94 | 5.72 |
|  | N_b_ ~ CV length * Treatment + (1 \| Lake) | − 79.96 | 179.56 | 10.33 |
| Sex Ratio | N_b_ ~ 1 + (1 \| Lake) | − 80.76 | 169.23 | 0.00 |
|  | N_b_ ~ Sex Ratio + (1 \| Lake) | − 80.51 | 172.09 | 2.87 |
|  | N_b_ ~ Treatment + (1 \| Lake) | − 80.57 | 172.22 | 3.00 |
|  | N_b_ ~ Sex Ratio + Treatment + (1 \| Lake) | − 80.34 | 175.68 | 6.45 |
|  | N_b_ ~ Sex Ratio * Treatment + (1 \| Lake) | − 80.05 | 179.74 | 10.52 |

**Conversion of *N_b_* to *N_e_* Using Two Life-History Traits**

We calculated $\hat{N}_{e(adj)}$ from $\hat{N}_{b}$ using age-at-maturity (α) and adult lifespan (AL) according to the formulae using two life-history traits in Waples et al. (2014). Age-at-maturity in our populations was calculated based on fish captured during stock assessment, using generalized linear mixed models with a binomial fit to estimate age-at-fifty-percent-maturity (A_50_, here we refer to as α). Adult lifespan was approximated by taking the maximum age (ω), subtracting α and adding 1 (Table B1). To calculate $\hat{N}_{e(adj)}$, we first took the raw $\hat{N}_{b}$ values and calculated adjusted $\hat{N}_{b}$ (or $\hat{N}_{b(adj)}$) as follows:

$$\hat{N}_{b(adj)}=\frac{raw \hat{N}_{b}}{1.103-0.245\times log(\frac{AL}{\alpha})}$$

Then, using $\hat{N}_{b(adj)}$, we calculated $\hat{N}_{e(adj)}$:

$$\hat{N}_{e(adj)}=\frac{\hat{N}_{b(adj)}}{0.485+0.758\times log(\frac{AL}{\alpha})}$$

We also calculated the ratio between $\hat{N}_{e(adj)}$ and $\hat{N}_{c}$ to assess whether genetic compensation was detected with the converted effective sizes.

$\hat{N}_{e(adj)}$ followed the same trends through time as $\hat{N}_{b}$, with only Mud having a significant change in $\hat{N}_{e(adj)}$ through time (indicated by non-overlapping confidence intervals; Figure B1). The ratio between $\hat{N}_{e(adj)}$ and $\hat{N}_{c}$ also had the same trends as with $\hat{N}_{b}$/$\hat{N}_{c}$, with all the harvest lakes increasing in the ratio through time, indicating that genetic compensation has occurred.

**Table S11**: Effective population size (N_e(adj)_) calculated from the effective number of breeders (N_b_) using age-at-fifty-percent-maturity (α) and adult lifespan (AL). N_b(adj)_ is the adjusted N_b_ based on the two life-history traits.

|  | Population - Year | α | AL | $\hat{N}_{b(adj)}$ | $\hat{N}_{e(adj)}$ |
| --- | --- | --- | --- | --- | --- |
| Control | Helen 2017 | 1.17 | 5 | 69.9 | 73.5 |
|  | Helen 2019 | 1.13 | 5 | 122.0 | 126.3 |
|  | Helen 2020 | 2.14 | 6 | 67.9 | 90.0 |
|  | Marg 2017 | 1.57 | 4 | 86.1 | 116.0 |
|  | Marg 2019 | 1.56 | 5 | 44.9 | 54.1 |
|  | Marg 2020 | 1.87 | 5 | 72.4 | 97.1 |
|  | McNair 2017 | 2.32 | 8 | 45.3 | 54.4 |
|  | McNair 2019 | 2.29 | 6 | 17.4 | 24.1 |
|  | McNair 2020 | 3.1 | 7 | 21.8 | 34.3 |
| Harvest | Mud 2017 | 1.38 | 6 | 61.3 | 64.8 |
|  | Mud 2019 | 0.77 | 5 | 26.4 | 23.6 |
|  | Mud 2020 | 1 | 3 | 21.8 | 25.8 |
|  | Olive 2017 | 2.04 | 5 | 49.7 | 70.6 |
|  | Olive 2019 | 1.46 | 4 | 34.4 | 44.3 |
|  | Olive 2020 | 1.68 | 4 | 53.4 | 75.3 |
|  | Temple 2017 | 3.29 | 7 | 25.7 | 42.7 |
|  | Temple 2019 | 2.44 | 8 | 68.1 | 84.0 |
|  | Temple 2020 | 2.76 | 10 | 44.8 | 53.0 |


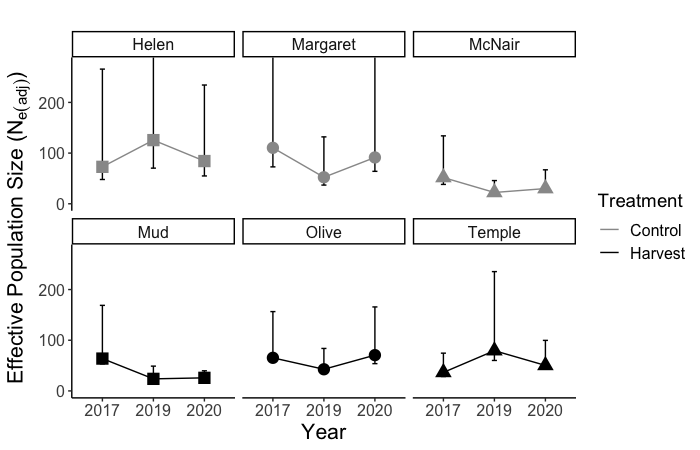


*

*

*

**Figure S1:** Effective population size (N_e(adj)_) through time in control and harvest populations of brook trout in the Rocky Mountains. Error bars indicate converted 95% confidence intervals. Asterisks indicate where the error bar did not fit within the limits of the graph.
